# Cell-Specific RNA Binding Protein Rbfox2 Regulates Ca_V_2.2 mRNA Exon Composition and Ca_V_2.2 Current Size

**DOI:** 10.1523/ENEURO.0332-16.2017

**Published:** 2017-10-10

**Authors:** Summer E. Allen, Cecilia P. Toro, Arturo Andrade, Eduardo J. López-Soto, Sylvia Denome, Diane Lipscombe

**Affiliations:** 1 Department of Neuroscience, and the Brown Institute for Brain Science, Brown University, Providence, RI 02912; 2 Department of Biology, Linfield College, McMinnville, OR 97128; 3 Department of Biological Sciences, University of New Hampshire, Durham, NH 03824

**Keywords:** alternative splicing, calcium channels, N-type, rbfox2, splicing factors, superior cervical ganglia

## Abstract

The majority of multiexon mammalian genes contain alternatively spliced exons that have unique expression patterns in different cell populations and that have important cell functions. The expression profiles of alternative exons are controlled by cell-specific splicing factors that can promote exon inclusion or exon skipping but with few exceptions we do not know which specific splicing factors control the expression of alternatively spliced exons of known biological function. Many ion channel genes undergo extensive alternative splicing including *Cacna1b* that encodes the voltage-gated Ca_V_2.2 α1 subunit. Alternatively spliced exon 18a in *Cacna1b* RNA encodes 21 amino acids in the II-III loop of Ca_V_2.2, and its expression differs across the nervous system and over development. Genome-wide, protein-RNA binding analyses coupled to high-throughput RNA sequencing show that RNA binding Fox (Rbfox) proteins associate with Ca_V_2.2 (*Cacna1b*) pre-mRNAs. Here, we link Rbfox2 to suppression of e18a. We show increased e18a inclusion in Ca_V_2.2 mRNAs: (1) after siRNA knockdown of Rbfox2 in a neuronal cell line and (2) in RNA from sympathetic neurons of adult compared to early postnatal mice. By immunoprecipitation of Rbfox2-RNA complexes followed by qPCR, we demonstrate reduced Rbfox2 binding upstream of e18a in RNA from sympathetic neurons of adult compared to early postnatal mice. Ca_V_2.2 currents in cell lines and in sympathetic neurons expressing only e18a-Ca_V_2.2 are larger compared to currents from those expressing only Δ18a-Ca_V_2.2. We conclude that Rbfox2 represses e18a inclusion during pre-mRNA splicing of Ca_V_2.2, limiting the size of Ca_V_2.2 currents early in development in certain neuronal populations.

## Significance Statement

Cell-specific alternative splicing of neuronal pre-mRNAs regulates development, axon targeting, neuronal excitability, circuit formation, G protein signaling, and drug action. Despite their importance, few studies have shown how specific splicing factors regulate exons of known biological function. We show that the RNA binding protein Rbfox2 represses cell-specific splicing of a highly conserved exon 18a in *Cacna1b*. By independent approaches, we show that e18a regulates the overall activity of Ca_V_2.2 channels in cell lines and in neurons. Cell-specific factors determine when and which Ca_V_ channel isoforms are expressed in a given cell type, our data establish Rbfox2 as a regulator of Ca_V_2.2 channel currents in neurons.

## Introduction

Alternative exon usage is a feature of nearly all multiexon genes, and it is extensive in the nervous system of several species including humans and mice (Vuong et al., 2016). This form of cell-specific pre-mRNA processing greatly expands the coding capacity of genes without increasing genetic load. The resulting pool of mRNA splice isoforms in specific tissues carries a rich source of information about the function and the state of the cell (Lipscombe and Andrade, 2015; Lipscombe et al., 2015). Cell-specific inclusion or exclusion of alternative exons in the nervous system is essential for development, axon targeting, neuronal excitability, circuit formation, G protein signaling, and drug action (Grabowski and Black, 2001; Xie and Black, 2001; Gray et al., 2007; Asadi et al., 2009; Jepson et al., 2013; Lipscombe et al., 2013a; Lipscombe et al., 2013b; Saito et al., 2016). Global genetic deletion of cell-specific splicing factors in mice often results in lethality, pointing to a critical role of splicing factors early in development (Ule et al., 2005; Gehman et al., 2011; Gehman et al., 2012). Knowing the cell-specific signals and proteins that control alternative exon usage is key to understanding the mechanisms that generate unique cell-specific patterns of mRNA isoforms and cell-specific functions (Schmucker et al., 2000; Aoto et al., 2013; Lah et al., 2014).

**Figure 1.**
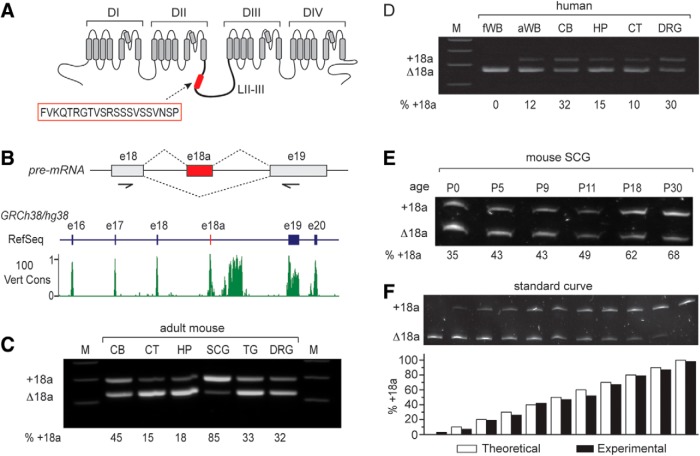
E18a expression in mouse and human tissue. ***A***, Transmembrane spanning and pore regions of a voltage-gated calcium (Ca_V_) channel α_1_ subunit. Highlighted are the four major domains (DI-DIV) and approximate location of the 21-amino acid peptide (red box) in the intracellular loop II-III (LII-III) encoded by e18a of *Cacna1b*. ***B***, upper panel, Schematic of Ca_V_2.2 pre-mRNA showing the e18a region and the two splice options (+18a or Δ18a). Arrows indicate the approximate location of PCR primers used to amplify human and mouse cDNAs. Lower panel, Visualization of RefSeq Genes and 100 vertebrates Basewise Conservation by PhastCons tracks using UCSC Genome Browser for human *CACNA1B* chr9:138 005,835-138 026,803 of Human December 2013 (GRCh38/hg38) assembly (https://genome.ucsc.edu/index.html; Kent et al., 2002). The region shown contains exons e16, e17, e18, e18a, e19, and e20 of human *CACNA1B.* In the RefSeq track, exons are denoted by vertical bars and introns by horizontal lines. E18a is not yet annotated in human genome assemblies, but we show its location by a red vertical bar. The conservation track displays the PhastCon scores from 0-1 after applying two-pixel smoothing. Regions of high conservation across vertebrates correspond to exons, including e18a. There is also a region of high conservation in the intron downstream of e18a. ***C***, ***D***, RT-PCR-amplified cDNA products separated in 3% agarose gels show e18a inclusion and exclusion in mRNAs of different tissue (upper band e18a, lower band Δ18a). Primers were located in constitutive e18 and e19, flanking e18a; product sizes were 291 bp (e18a) and 228 bp (Δ18a). ***C***, Adult mouse cortex (CT) and hippocampus (HP) show the lowest levels of e18a inclusion, adult SCG the highest, and adult CB, trigeminal ganglia (TG), and DRG have intermediate levels of e18a inclusion. ***D***, Human fetal whole brain (fWB) lacked detectable e18a, adult human whole brain (aWB), CT, and HP had low levels of e18a; and ∼30% of product amplified from adult human cerebellum (CB) and DRG contained e18a. ***E***, RT-PCR amplified cDNA products from mouse SCG RNA, separated in 8% denaturing polyacrylamide gel. E18a inclusion increases with development from P0 (35%) to P30 (68%). ***F***, Calibration curve validates quantification of relative amounts of e18a and Δ18a by RT-PCR. PCR products were separated in denaturing 8% polyacrylamide. Bar graph shows % e18a-Ca_V_2.2 cDNA clone in the mix (white), and % e18a measured from densitometry analysis (black). Theoretical and experimental values are similar.

All mammalian *Cacna1* genes contain alternatively spliced exons that are included or excluded in different combinations depending on cell type and stage of development (Lipscombe et al., 2013a; Lipscombe and Andrade, 2015). The expression pattern and function of several alternatively spliced exons of *Cacna1b* are relatively well characterized; they are differentially expressed according to tissue and cell type and/or development, and their inclusion impacts specific aspects of channel function (Raingo et al., 2007; Andrade et al., 2010). Here, we focus on an alternative exon in *Cacna1b*, e18a, one of four in this gene that affect Ca_V_2.2 channel function. The prevalence of e18a-containing Ca_V_2.2 mRNAs varies with nervous system region, and is higher in tissues of adult compared to newborn rats (Pan and Lipscombe, 2000; Gray et al., 2007). E18a encodes 21 amino acids in the II-III loop of Ca_V_2.2 (Fig. 1*A*), and e18a-containing Ca_V_2.2 mRNAs increase in certain cell populations during development in rat (Pan and Lipscombe, 2000). E18a-Ca_V_2.2 channels are less sensitive to cumulative inactivation compared to e18a-lacking (Δe18a-Ca_V_2.2) channels, but only when Ca_V_2.2 is coexpressed with accessory subunits Ca_V_β_1_ or Ca_V_β_4_, not Ca_V_β_2_ or Ca_V_β_3_ (Pan and Lipscombe, 2000; Thaler et al., 2004; Gray et al., 2007). While Ca_V_2.2 channels can associate with all four Ca_V_β subunits in expression systems, >50% of Ca_V_2.2 protein in the brain is in a complex with Ca_V_β_3_ and Ca_V_β_4_ (Castellano et al., 1993; Altier et al., 2002; Müller et al., 2010) and in sympathetic neurons, Ca_V_β_2_ and Ca_V_β_3_ subunits associate with functional Ca_V_2.2 channels (Namkung et al., 1998).

There are four well-characterized sites of tissue-specific alternative splicing in *Cacna1b* and each has a distinct expression profile (e18a, e24a, e31a, and e37a/e37b). The brain-specific RNA binding protein Nova2 regulates the splicing of cassette exons e24a (promotes inclusion) and e31a (promotes exclusion; Ule et al., 2003; Allen et al., 2010), and the RNA binding protein Rbfox2 has been shown to bind Ca_V_2.2 pre-mRNA close to the alternatively spliced e18a (Gehman et al., 2012; Weyn-Vanhentenryck et al., 2014). Here, we show that Rbfox2 suppresses e18a inclusion in a neuronal cell line, and in early postnatal sympathetic neurons. By two independent approaches we found that e18a supports larger Ca_V_2.2 currents in (1) cell lines expressing cloned channels, and (2) sympathetic neurons from mice genetically modified to restrict exon choice. Our findings show that Rbfox2 regulates Ca_V_ ion channel activity through cell-specific control of exon selection according to cell type and development.

## Materials and Methods

### SCG cell isolation and culture

Superior cervical ganglia (SCG) were removed from postnatal P0-P2 mice. We dissociated ganglia in HBSS (CaCl_2_, MgCl_2_, MgSO_4_) containing 1 mg/ml trypsin (TRL3, Worthington) while incubating in a 37°C water bath for 45-60 min. Following trypsinization, cells were dissociated by 5 min of trituration using a fire-polished pipette. Cells were plated on coverslips coated with laminin (Invitrogen). Neurons were maintained at 37°C with 5% CO_2_. Twenty-four hours after plating, cells were treated with 10 µM ara-C (Sigma) to inhibit glia growth.

### Clones and transfection

tsA201 cells were grown in DMEM (Sigma) +10% fetal bovine serum (Gibco) and split when 70% confluent. Cells were transfected when 70% confluent using Lipofectamine 2000 (Invitrogen) and Opti-MEM (Sigma). Cells were transfected with cDNA clones of Ca_V_2.2, Ca_V_β_2a_ or Ca_V_β_3_, and Ca_V_α_2_δ_1_ in a molar ratio of 1:1:1 along with EGFP. The following clones, which were isolated in our lab from rat brain, were used in these experiments: Ca_V_β_3_ (M88751); Ca_V_α_2_δ_1_ (AF286488); Ca_V_2.2[Δ18a,37b] (AF055477); and Ca_V_2.2[18a, 37b] (HQ008360). The only sequence difference among Ca_V_2.2 cDNA clones used in this study is whether e18a-encoding sequence is absent or present. All other splice sites including e37b are identical. The Ca_V_β_2a_ clone was a gift from David Yue. It was subcloned into pcDNA3.1/(zeo+) using EcoRI.

### Cross-linking followed by immunoprecipitation (CLIP) protocol for Rbfox2

Two SCG were extracted from each of 10 postnatal P8-P11 and 10 adult mice (5 males and 5 females), snap frozen in dry ice, and then transferred to liquid N_2_. Following cross-linking in 1% formaldehyde in PBS for 10 min, the reaction was quenched with 125 mM glycine for 5 min [all at room temperature (RT)]. Samples were washed three times in chilled PBS and were resuspended in lysis buffer (50 mM HEPES-KOH at pH 7.5, 140 mM NaCl, 1 mM EDTA, 1.0% Triton X-100, 0.1% sodium deoxycholate, 0.1% SDS and 1× protease inhibitors) and then passed through a pipette (20×) and vortexed (3×). Samples were sonicated (Q500A, Qsonica) 10 × 30 s at highest amplitude pulses separated by 1-min rests, and the temperature was maintained at 4°C. Following a 8 min centrifugation at 10,000 × *g* at 4°C, supernatants were incubated at 37°C for 30 min with excess DNase I (0.5 U/µl, Ambion) and RNase OUT (1 U/µl, Invitrogen). Samples were diluted to a final volume of 500 µl in dilution buffer (16.7 mM Tris-HCl, pH 8.0, 1.2 mM EDTA, 2.2% Triton X-100, 0.01% SDS, 334 mM NaCl, and 1× protease inhibitor). A 5% sample was used as the input control. Protein-RNA complexes were isolated as follows: immunoprecipitated overnight at 4°C with 5 µg of polyclonal RBM9 antibody (catalog number A300-864A, Bethyl) or 5 µg of normal rabbit IgG (catalog number 2729, Cell Signaling), incubated at 4°C with gentle rotation for 2 h with prewashed 50 µl Magna ChIP Protein A + G Magnetic Beads (catalog number 16-663, Millipore), unbound material was removed by sequential washes of 4 min each at 4°C in low salt buffer (20 mM Tris-HCl, pH 8.0, 150 mM NaCl, 2 mM EDTA, 0.1% SDS, and 1% Triton X-100), high salt buffer (20 mM Tris-HCl, pH 8.0, 500 mM NaCl, 2 mM EDTA, 0.1% SDS, and 1% Triton X-100), LiCl buffer (10 mM Tris-HCl, pH 8.0, 1 mM EDTA, 250 mM LiCl, 1% NP40, and 1% sodium deoxycholate), and TE. Finally, RNA was extracted using TRIzol Reagent according to the manufacturer’s instructions (Invitrogen/ThermoFisher Scientific). Glycogen was used as a carrier to increase RNA yield precipitation. All solutions were prepared in RNase-free H_2_O.

### RT-qPCR

First strand cDNA was generated by random primers (250 ng) using SuperScript III First-Strand Synthesis System (Invitrogen/ThermoFisher Scientific) from immunoprecipitated and input RNA samples isolated from sympathetic ganglia of postnatal P8-P11 and adult mice. A total of 1 µl of cDNA template was amplified by real-time PCR in a 10-µl reaction using *Cacna1b*-specific primers (300 nM) that flank an Rbfox protein binding site upstream of e18a: JLS27-5’ GCCACGTGTCACTCATGTCT/JLS28-5’ CCTCGAGTTTGCTTTACAAAA and; a downstream sequence of e18a similar to Rbfox protein binding site: JLS29-5’ TCCACTCCTGACTGCTTTCC/JLS30-5’ TGCATAGGACTTGGAGACAGC. Each sample was run in triplicate using Power SYBR Green PCR Master Mix (catalog number 4368706, ThermoFisher Scientific) and H_2_O in a StepOnePlus Real-Time PCR System (Applied Biosystems). PCRs involved an initial 10 min of incubation at 95°C, 45 cycles of 20 s at 95°C, and 1 min at 60°C. We estimated real-time PCR efficiencies (Pfaffl, 2001) from serial dilutions of gDNA containing target sequences and using nine replicates per DNA sample, these were 93.44% and 93.46% for primers to regions upstream and downstream of e18a, respectively. PCR specificity was confirmed by PCR product length and using melting curve analysis after the PCR. Melting curves were obtained, following 15 s at 95°C, by a temperature gradient from 60°C to 95°C at +0.3°C/step. Ct values were calculated from input and IP sample templates. Each dataset was generated from three qPCRs for each of three different biological samples, and normalized to rabbit IgG IP values. The % of input method was used to quantify Rbfox2 binding levels to 5’ and 3’ e18a regions in *Cacna1b* sympathetic ganglia of P8-P11 and adult mice.

### Electrophysiology

#### tsA201 cells

We performed standard whole-cell patch clamp recording. Extracellular solution: 135 mM choline Cl, 1 mM CaCl_2_, 4 mM MgCl_2_, and 10 mM HEPES, pH 7.2 with CsOH. Internal: 126 mM CsCl, 10 mM EGTA, 1 mM EDTA, 10 mM HEPES, and 4 mM MgATP, pH 7.2 with CsOH.

#### SCG neurons

External solution: 135 mM TEA-Cl, 4 mM MgCl_2_, 10 mM CaCl_2_, 10 mM HEPES, and 100 nM TTX, pH 7.2 w/TEA-OH. Internal: 126 mM CsCl, 1 mM EDTA (Cs_2_), 10 mM EGTA (Cs_4_), 10 mM HEPES, and 4 mM MgATP, pH 7.2 with CsOH. We sylgard-coated and fire-polished electrodes to a resistance of 2-3 MΩ (for tsA201 cells) or 4–6 MΩ (for SCG neurons).

#### Data analysis

We used pClamp version 10 software and the Axopatch 200A (Molecular Devices) for data acquisition; data were filtered at 2 kHz (–3 dB) and sampled at 20 kHz. We compensated series resistance by 70–90% with a 7-μs lag and performed online leak correction with a P/–4 protocol. All recordings were obtained at RT. To measure current amplitudes, we either isolated the Ca_V_2.2 component by ω-conotoxin GVIA (2 μM) subtraction or we used a combination of inhibitors of Ca_V_1 and Ca_V_2.1 currents with a mixture of isradipine (10 μM) and ω-agatoxin IVA (50 nM). For the ω-conotoxin GVIA (2 μM) subtraction method, we first recorded the whole-cell Ca_V_ current, inhibited the Ca_V_2.2 component, and subtracted the resistant current (non-Ca_V_2.2) from the whole-cell Ca_V_ current to isolate the Ca_V_2.2 current (Andrade et al., 2010). All cells were maintained in a NaCl external solution (135 mM NaCl, 4 mM MgCl_2_, 10 mM CaCl_2_, and 10 mM HEPES, pH 7.2 w/NaCl-OH) and, after whole-cell formation, cells were lifted and placed in the stream of a TEA-Cl based solution applied via a microperfusion system (VC-M6, Warner Instruments).

### Genomic sequence alignment

We used the BLAT alignment tool in the UCSC genome browser to find sequences analogous to mouse e18a in the *Cacna1b* genes of other species. For each species, we identified the 18a exonic sequence as well as 200 nt of each of the neighboring introns. We then used the alignment tool on Vector NTI Advance 10 (Invitrogen) to align the sequences from all species.

### RT-PCR

We dissected SCG from wild-type mice (4-10 mice per age group). Ganglia were homogenized in TRIzol (Invitrogen) using a Polytron homogenizer. We used SuperScript II (Invitrogen) to reverse transcribe 1 µg of RNA per group. We used 1 µl of this first-strand cDNA in a 25-µl PCR containing Taq polymerase (New England Biolabs). E18a RT-PCR primers are: BU2118 5’ GGCCATTGCTGTGGACAACCTT and BD2324 5’ CGCAGGTTCTGGAGCCTTAGCT. PCR was performed with a thermocycler (Bio-Rad DNAEngine), using the following program: 1 cycle of 95°C for 2 min; 25 cycles of 95°C for 0.5 min, 60°C for 0.5 min, 72°C for 1 min, a final 6-min extension step at 72°C. The RT-PCR protocol for amplifying cDNA from F11 cells was identical. RT-PCR products were run on 8% denaturing polyacrylamide gels. Gels were stained with SYBR Gold stain (Invitrogen). Unsaturated bands were imaged on a Bio-Rad gel doc station. We used the program Scion Image to analyze unsaturated band densities. Human total RNA samples were purchased from Clontech. Each sample contains pooled total RNA from at least four individuals. Lot numbers are indicated between parenthesis: whole brain (1402002), fetal brain (1412624A), cerebellum (CB; 1506001A), hippocampus (HP; 1002006), dorsal root ganglia (DRG; 1505766A). We used iScript RT (Bio-Rad) to reverse transcribe 1 μg of total RNA per sample. We used 1 μl of cDNA in a 20-μl PCR with AmpliTaq Gold 360 Master Mix (ThermoFisher). We used human-specific primers: F, 5’ TGGAAGAAGCAGCCAATCAG; R, 5’ CCAGGTGCGTCTTCATGTC. PCR was performed using the thermocycler CT-1000 (Bio-Rad) with the following conditions: 1 cycle of 95°C degrees for 10 min; 30 cycles of 95°C degrees for 30 s, 57°C for 30 s, and 72°C for 45s. 1 cycle of 72°C for 7 min. RT-PCR products were run in 3% agarose gels stained with ethidium bromide. Densitometry analysis of unsaturated bands was performed with ImageJ.

### F11 cell culture and siRNA transfection

F11 cells were split twice each week and were grown in DMEM culture media plus 10% fetal bovine serum and 1% penicillin/streptomycin. One day before transfection, cells were plated in six-well plates without antibiotics. We used Lipofectamine 2000 (Invitrogen) to transfect cells with siRNAs for 48-60 h. siRNAs used (Thermo Scientific Dharmacon): Rbfox2 (original sequence provided by Dr. Douglas Black, UCLA): 5’ CCUGGCUAUUGCAAUAUUUUU. 5’ Fox-2b: UAUUGCAAUAGCCAGGCCUCUU. siGENOME Cyclophilin β control siRNA: catalog number D-001136-01-05. siGENOME nontargeting siRNA #3: catalog number D-001210-03-05.

### Western blotting

We used Lipofectamine 2000 (Invitrogen) to transfect 10 cm dishes of F11 cells with 0.5 µg EGFP (C1, Clontech) alone, EGFP + 6 µg Rbfox2 plasmid (provided by Douglas Black’s lab; used as a size control), or EGFP + 100 nM Rbfox2 siRNA for 48 h. Media were changed after 6 h. We estimate that ∼25% of cells were transfected with EGFP. RIPA buffer containing protease inhibitors (Roche) was used to lyse the cells. Lysed cells were spun at 13,500 rpm at 4°C for 20 min. We used a BCA kit (Pierce) to determine the protein concentration of the supernatant. We denatured 12 µg of each sample for 10 min at 90°C and then ran samples on a 4% stacking and 15% resolving SDS-PAGE gel. Protein was transferred to a nitrocellulose membrane (Protran, Whatman) and blocked with 5% dry milk in PBS with 0.1% Tween 20 (PBST) for 1 h at RT. The blot was then incubated in a rabbit anti-Rbfox RRM primary antibody that recognizes all Fox proteins (1:2000 in PBST with 1% BSA, gift from Douglas Black) for 1 h at RT. The blot was rinsed and then incubated in a donkey-anti-rabbit horseradish peroxidase secondary (1:7500 in PBST with 1% BSA, Jackson ImmunoResearch) for 1 h at RT. Proteins were detected with an ECL chemiluminescence kit (Vector). The blot was then stripped with 2-mercaptoethanol and SDS and reprobed with a rabbit anti-GAPDH antibody (1:1000 in PBST with 1% BSA, Cell Signaling).

### SCG injections

SCG neurons used for electrophysiology were injected 24 h after plating; those used for immunocytochemistry were injected 7 d after plating. We used an InjectMan NI2 manipulator and Femtojet microinjector (Eppendorf) for injections. We combined 10 µl of 20 µM siRNA with 10 μl of 8.9 mg/ml dextran fluorescein (Invitrogen) and spun down any undissolved dextran by spinning the sample at 14,000 rpm for 10 min at 4°C. We then used 2 µl of this solution to load the microinjection tip (Eppendorf). Injection parameters: Pi 150 hPa, Ti 0.4 s, Pc 40 hPa.

### Immunocytochemistry

SCG neurons were fixed with 4% paraformaldehyde in PBS for 15 min. We then blocked the cells for 1 h at RT in 10% normal goat serum (NGS) in PBS. Cells were incubated in a rabbit anti-Rbfox2 antibody (1:500, Bethyl) for 2.5 h at RT in 3% NGS. Cells were washed in PBS and then incubated in a goat-anti-rabbit rhodamine secondary antibody (1:200, Jackson ImmunoResearch) in 3% NGS for 45 min at RT. Images were captured with a confocal microscope (LSM 510, Zeiss), and analyzed with AxoVision 4.8.1 (Carl Zeiss Imaging Solutions).

### Mouse lines with limited exon choice

The Δ18a-Ca_V_2.2 targeting construct was created from Sanger BAC clone BMQ175b12 (Adams et al., 2005). Approximately 12-kb XbaI of mouse 129S genomic DNA was cloned into pBluescript II KS (-). Sequential site-directed mutagenesis PCR Stratagene's QuikChange II XL Site-Directed Mutagenesis kit (#20051) were used to remove e18a and its flanking introns, creating a precise fusion of e18 and e19. A loxP-NeoR-loxP cassette was PCR amplified from pL452 (PTA-4997) and inserted into the SpeI site of the intron between e17 and e18. Finally, ∼3-kb XbaI-KpnI containing e20 from BAC clone BMQ175b12 was added to increase the total genomic DNA in the final targeting construct to ∼11 kb. To create the e18a-Ca_V_2.2 targeting construct, site-directed mutagenesis was used to insert e18a into the Δ18a-Ca_V_2.2 targeting construct precisely between e18 and e19, resulting in a final e18/e18a/e19 targeting construct of ∼11.1 kb. Both targeting constructs were linearized with *Pvu*I for electroporation into mouse ES129 cells, and selected for NeoR. NeoR positive clones were confirmed by PCR and southern hybridization to contain the mutations of interest. Confirmed clones were microinjected into FVB/N embryos, and the resulting chimeras were mated with C57BL/6J to generate F1s. To delete NeoR, the F1 were mated to a Cre recombinase line (B6.FVB-Tg(EIIa-cre)C5379Lmgd/J, The Jackson Laboratory stock number 003724). Neo^-^ siblings were mated to produce homozygous animals in a mixed genetic background. These lines were subsequently backcrossed to C57BL/6 (Charles River) for at least six generations, to create lines in a C57BL/6 genetic background. The new strains will be designated *Cacna1b^tm3.1Dili^* (+18a) and *Cacna1b^tm4.1Dili^* (Δ18a) in the mouse genome informatics database (MGI). Mouse strains were viable and fecund see Table 1.

### Genotyping

#### Genotyping of Δ18a mice

These mice were genotyped using PCR amplification of genomic DNA extracted from tail biopsies. Two independent reactions were used. PCR 1: 5’ GTGGCTTCTAGCACAAACACG and 5’ CTCGGTGCTTTCTGTCCTGTCC amplified a region spanning from the intron upstream of e18 to e19, a 777-bp band is expected for Δ18a homozygous mice. PCR 2: 5’ GCAGCAGAGGTTCTGTTTGC and 5’ CTCGGTGCTTTCTGTCCTGTCC amplified a region spanning from the intron upstream of e19 and e19, a 880-bp band is expected from a Δ18a heterozygous mice mouse. No amplification is expected from homozygous mice. Both PCR conditions were performed with Taq DNA polymerase (NEB, M0267) with the following conditions: 94°C 2 min, 35 cycles (94°C 30 s, 60°C 30 s, 72°C 1 min), 72°C 10 min.

#### Genotyping of +18a mice

Similar to Δ18a mice, two independent PCRs were used. PCR 1: 5’ GTGGCTTCTAGCACAAACACG and 5’ CTCGGTGCTTTCTGTCCTGTCC amplifies a region that spans within e18a to e19, for homozygous mice a 840-bp product is expected. PCR was performed with Taq DNA polymerase (NEB, M0267) as follows: 94°C 2 min, 35 cycles (94°C 30 s, 62°C 30 s, 68°C 1 min), 68°C 10 min. PCR 2: 5’ CTGTTACTCCTGTCAGCTGG and 5’ CTCGGTGCTTTCTGTCCTGTCC amplifies a region that spans from intron upstream of e19 through e19. A 590-bp product is expected for wild-type and heterozygous mice, no product is expected for homozygous mice. This PCR was performed using Advantage 2 Polymerase (Clontech, 639201) as follows: 94°C 2 min, 35 cycles (94°C 30 s, 62°C 30 s, 68°C 1 min), 68°C 10 min.

### Statistical analysis of cumulative probability

Cumulative probability plots were constructed using a similar bin size for both conditions (see figure legends). We used horizontal lines to construct the plots. Cumulative probability plots were fit with a logistic function y = A/(1 + e ^–k(x-x0)^). In all cases *r*^2^ > 0.95 was obtained. Statistical tests are noted in text and described in more detail in Table 2.

## Results

### E18a expression patterns in different regions of the nervous system are similar in mouse, human, and rat

The amino acid composition of the Ca_V_ channel II-III linker differs across the major mammalian Ca_V_α_1_ subunits (Catterall, 2011; Lipscombe et al., 2013a) but exons e17, e18, and e18a, which encode the proximal region of the II-III linker, are conserved among mammalian *Cacna1b* orthologs (see Vertebrate Multiz Alignment & Conservation, 100 Species, track of the UCSC Genome Browser; Fig. 1*B*). E18a is an alternatively spliced cassette exon present in all mammalian *Cacna1b* genes but mRNAs containing this exon are frequently overlooked because e18a-Ca_V_2.2 mRNAs are most abundant in regions of the nervous system that have poor representation in the RefSeq database (Lipscombe et al., 2015; e.g., SCG (Fig. 1*C*) and spinal cord; Gray et al., 2007), and they are found in low abundance in regions with strong representation in the RefSeq database (e.g., cortex, CT; Fig. 1*C*,*D*; Gray et al., 2007). E18a is not yet annotated in RefSeq *Cacna1b* gene tracks (RefSeq; Fig. 1*B*) but it (and other conserved cell-specific alternatively spliced exons) can be resolved as a region of high conservation in multiple species alignments and conservation tracks (100 Vert Cons; Fig. 1*B*), and validated by analyses of mRNAs in specific cells and tissues (see below; Lipscombe et al., 2015).

Previous analyses of e18a-containing Ca_V_2.2 mRNA expression were performed primarily in rat tissues. To establish broader biological relevance, we analyzed e18a- and Δ18a-Ca_V_2.2 mRNAs in different regions of the mouse and human nervous systems (Fig. 1*C-F*). We used RT-PCR to amplify e18a or Δ18a-Ca_V_2.2 sequence (approximate location of primers shown in Fig. 1*B***)** from three different brain regions and three ganglia of adult mice (Fig. 1*C*). The relative intensity of the e18a (upper) and Δ18a (lower) bands differed across tissues; the strongest e18a signal was observed in SCG and the weakest in CT (Fig. 1*C*). The profile of e18a expression by RT-PCR analyses of human RNA was similar to findings in mouse when comparing homologous regions of the nervous systems (cerebellum, hippocampus, cortex, and DRG; Fig. 1, compare *C*, *D*). Furthermore, the enrichment of Ca_V_2.2 mRNAs containing e18a in SCG of adult mice is consistent with an increase in e18a-Ca_V_2.2 mRNA levels in these ganglia during development (Fig. 1*C*,*E*; Gray et al., 2007). E18a-Ca_V_2.2 mRNAs increased relative to Δ18a-Ca_V_2.2 mRNAs in SCG of mice from postnatal day 0 to postnatal day 30 (P0-P30) (4-10 mice pooled per group; Fig. 1*E*,*F*), similar to analyses of rat ganglia (Gray et al., 2007). Our analyses of human and mouse RNA confirms and extends prior studies in rat, and show that expression of e18a-Ca_V_2.2 mRNAs is tissue-specific and developmentally regulated. The similar expression profile across species suggests that cell-specific mechanisms that control e18a inclusion during alternative splicing of Ca_V_2.2 pre-mRNAs may be shared across rat, mouse, and human.

### Rbfox2 represses e18a expression in F11 neuronal cell line

**Figure 2.**
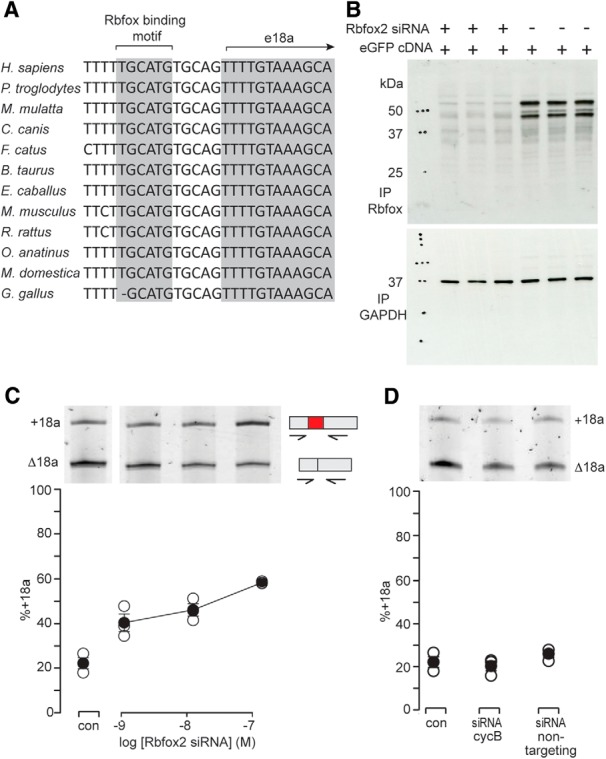
The splicing factor Rbfox2 represses e18a inclusion in a cell line. ***A***, Genome alignments: Rbfox binding motif (U)GCAUG located upstream of e18a (Minovitsky et al., 2005). Putative Rbfox consensus sequence is conserved across all 12 species shown. ***B***, Western blotting shows Rbfox protein in F11 cells transfected with siRNA against Rbfox2 (lanes 1-3) compared to control (lanes 4-6). Anti-Rbfox recognizes RNA binding motif (RRM) of all Rbfox protein isoforms (Gehman et al., 2011). The anti-GAPDH signal from the same membrane stripped and reprobed is shown. The experiment was run in triplicate, lanes 1-3 (siRNA) and lanes 4-6 (control). ***C***, RT-PCR analysis of +18a and Δ18a Ca_V_2.2 mRNAs from F11 cells transfected with Rbfox2-specific siRNA. Three independent transfections were analyzed per condition. E18a RT-PCR signal, as a percentage of total, was: 22.5 ± 2.3% (nontransfected F11) compared to 58.4 ± 2.5% in cells transfected with 100 nM siRNA to Rbfox2 (Student’s unpaired *t* test, *p* = 0.000129^a^). ***D***, RT-PCR analysis of Ca_V_2.2 mRNA extracted from nontransfected F11 cells (con), F11 cells transfected with siRNA to cyclophilin B (cycB siRNA), or transfected with nontargeting siRNA. Representative gel is shown with average % e18a signal in PCR amplification of F11 RNA from three experiments. Average +18a signals were similar: 22.5 ± 2.3% (con); 20.3 ± 2.3% (cycB); and 26.0 ± 1.7% (nontargeting siRNA; one-way ANOVA, *F* = 1.789, *p* = 0.246^b^).

Several lines of evidence suggest that Rbfox2 binds close to e18a: our alignment of nucleotide sequences at the intron-exon boundary of e18a from several *Cacna1b* genes reveals a putative Rbfox protein binding motif, (U)GCAUG, a few nucleotides from the start of e18a in the 5’ intron (Fig. 2*A*); genome-wide cross linking immunoprecipitation (CLIP) analyses of Rbfox2 binding to mouse brain RNA also identify a Rbfox2 binding site close to e18a in *Cacna1b* (Gehman et al., 2012; Weyn-Vanhentenryck et al., 2014); and RT-PCR analyses of e18a in RNA from P30 brains of Rbfox2 knockout mice show it is expressed at levels ∼ 10% higher compared to wild-type mouse brain (Gehman et al., 2012). When Rbfox proteins bind upstream of target exons they typically repress exon inclusion (Licatalosi et al., 2008; Yeo et al., 2009; Gehman et al., 2011; Damianov et al., 2016). We therefore used siRNA against Rbfox2 (Gehman et al., 2011) to test if Rbfox2 represses e18a inclusion in the F11 neuronal cell line (Fig. 2*B*,*C*). We used the F11 cell line (DRG/neuroblastoma fusion) because it expresses endogenous Ca_V_2.2 and Rbfox2, and these cells are relatively easy to transfect with high efficiency, facilitating biochemical and RT-PCR analyses.

We confirmed the efficacy of Rbfox2 siRNA in F11 cells by Western blot analysis (Fig. 2*B*). Using an antibody that recognizes the RNA binding motif of all Rbfox proteins (Gehman et al., 2011), we found almost complete loss of anti-Rbfox signal in F11 cells transfected with Rbfox2 siRNA and EGFP cDNA (Fig. 2*B*, lanes 1-3) compared to cells transfected only with EGFP cDNA (Fig. 2*B*, lanes 4-6). The Rbfox antibody recognized two major bands and one minor band that correspond to endogenous Rbfox2 protein (Fig. 2*B*, lanes 4-6) based on their sensitivity to Rbfox2 siRNA (Fig. 2*B*, lanes 1-3). Our findings are consistent with other reports of different size Rbfox2 proteins in Western analysis that likely correspond to different isoforms of Rbfox2 (Nakahata and Kawamoto, 2005; Baraniak et al., 2006; Chen and Manley, 2009; Damianov and Black, 2010).

To test our hypothesis that Rbfox2 represses e18a inclusion in F11 cells, we RT-PCR amplified from RNA derived from these cells, using Ca_V_2.2-specific primers that flanked e18a (Fig. 2*C*). We found a Rbfox2 siRNA concentration-dependent increase in Ca_V_2.2 mRNAs containing e18a in cells transfected with 100 nM Rbfox2 siRNAs (240% increase compared to nontransfected cells, Student’s unpaired *t* test, *p* = 0.000129^a^). siRNA designed against a different region of the Rbfox2 gene also increased e18a inclusion but with lower efficiency (64% increase, data not shown). In contrast, the relative abundance of Ca_V_2.2-e18a and Ca_V_2.2-Δ18a mRNAs was not consistently different in cells transfected with siRNA against cyclophilin B (a housekeeping gene) or a control nontargeting siRNA (#3 from Dharmacon) when compared to nontransfected cells (Fig. 2*D*; one-way ANOVA, *p* = 0.246^b^).

### Rbfox2 binding upstream of e18a *Cacna1b* RNA is lower in adult

**Figure 3.**
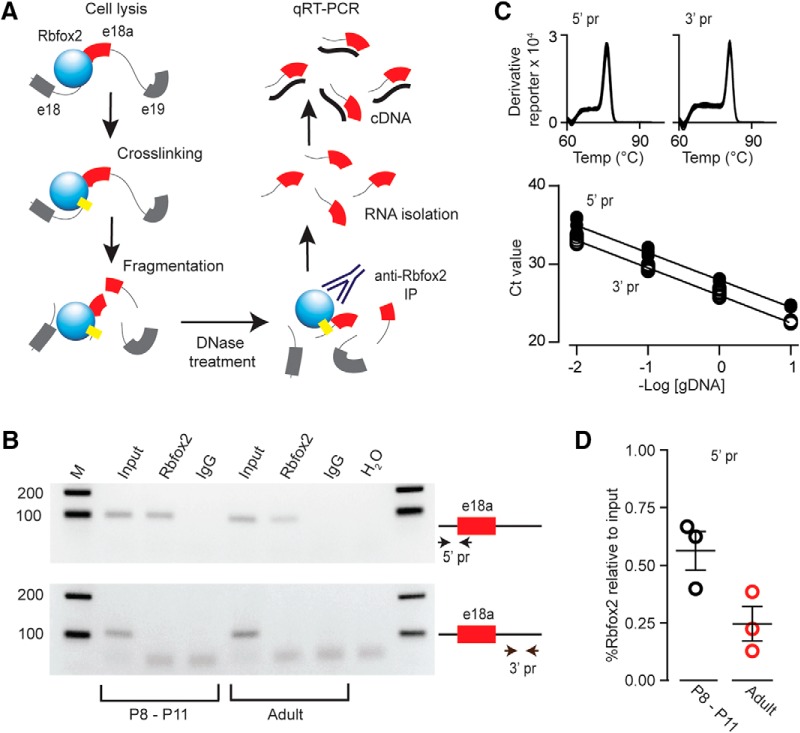
Rbfox2 binding to Ca_V_2.2 pre-mRNA is reduced in adult compared to P8-P11 sympathetic ganglia. ***A***, CLIP steps to quantify Rbfox2 binding to its motif in the intron 5’ to e18a in Ca_V_2.2 pre-mRNA *in vivo*. Cell lysis, protein cross-linking, nucleic acid fragmentation, DNase treatment to remove gDNA, Rbfox2 immunoprecipitation with polyclonal RBM9 antibody, RNA isolation from the immunocomplex, and first strand RT followed by qPCR. ***B***, CLIP-RNA samples from SCG of postnatal (P8-P11) and adult mice were used for endpoint RT-PCR. Endpoint PCR products were amplified from first strand cDNA using two sets of primers, one set flanks sequence 5’ to e18a containing the Rbfox2 binding motif (5’ pr-e18a; upper gel) and another other one flanking a sequence 3’ to e18a (3’ pr; lower gel) was used as control. Upper gel, 5’-e18a primers amplify 99-bp products from postnatal and adult samples after DNase treatment both before (input; lane 2) and after Rbfox2-IP (Rbfox2; lane 3). Lower gel, 3’-e18a primers amplify 108-bp products from postnatal (P8-P11) and adult samples after DNase treatment before (input; lane 5) but not after Rbfox2-IP (Rbfox2; lane 6). 5’-e18a and 3’-e18a primers did not amplify products from IgG or H_2_O control samples (lanes 4, 7, and 8). Products were separated in 2% agarose gel (one biological triplicate, three mice each condition). Size markers are shown (bp; lanes 1 and 9). ***C***, Melting and standard curves for 5’-e18a and 3’-e18a qPCRs establish the quality and efficiency of the two primer sets used for qPCR data in panel ***D***, Melting curves for 5’-e18a and 3’-e18a PCR products were generated after the qPCR using a temperature gradient from 60°C to 95°C (+0.3°C/step). The single sharp peak corresponds to a single product. Standard curves show Ct values for each qPCR primer set and establish they have the same efficiency (slopes = -3.48 ± 0.07 and -3.51 ± 0.05 for 5’-e18a and 3’-e18a qPCRs, respectively). ***D***, Percentage input method (Haring et al., 2007) used to quantify Rbfox2-IP RNA template by 5’-e18a qPCR and referenced to input before Rbfox2-IP. Each point represents a sample containing six SCG from three mice. The complete experiment (from SCG harvesting to qPCR) was performed in triplicate for each age group (P8-P11 and adult). Mean ± SE in % Rbfox2 relative to input for adult: 0.1872 ± 0.05; P8-P11: 0.5471 ± 0.07, *p* = 0.02^c^. Mean and SE values are indicated by horizontal bars.

E18a is expressed at particularly high levels in sympathetic neurons of adult SCG, but early in development e18a levels are low (∼35% at P0, ∼40% at P8-P10 increasing to ∼85% in adult; Fig. 1). To determine if Rbfox2 protein regulates e18a expression in neurons, we quantified levels of Rbfox2 bound to its putative binding site upstream of *Cacna1b* e18a in RNA isolated from SCG of postnatal (P8-P10) and adult mice. We used cross linking immunoprecipitation (CLIP) with anti-Rbfox2, and then RT-qPCR (Fig. 3*A*). Two primer pairs were used to amplify regions of similar size from Ca_V_2.2 pre-mRNA: one contains the TGCATG Rbfox2 binding motif in the intron 5’ to e18a (Fig. 2*A*) and the second primer pair amplifies a region in the intron 3’ to e18a containing a sequence GGCATG of unknown function, but which differs from the Rbfox2 motif by 1 nt (Fig. 3*B-D*). The complete CLIP-RT-qPCR procedure was performed in biological triplicate using three mice (six SCG) per biological sample for each age group. Primers amplified the expected size products from preimmunoprecipitated samples (input), but only 5’-e18a primers, not 3’-e18a primers, amplified product from samples following immunoprecipitation with anti-Rbfox2 (Rbfox2; Fig. 3*B*). No products were amplified in IgG and water control samples (Fig. 3*B*). We were careful to optimize conditions to achieve high efficiency and high specificity for both primer pairs (see Materials and Methods; Fig. 3*C*). The input signal was used as the reference for qPCR normalization (Pfaffl, 2001) and, as shown, Rbfox2 bound significantly more 5’-e18a intron template in P8-P11 samples compared to adult (Fig. 3*D*, Student’s unpaired *t* test, *p* = 0.02^c^). Our data provide direct evidence of greater Rbfox2 binding in the 5’-e18a intron of Ca_V_2.2 pre-mRNA in SCG in young postnatal animals as compared to adult. Our data are consistent with Rbfox2 acting to suppress e18a inclusion during alterative splicing of Ca_V_2.2 pre-mRNA early in development.

### Rbfox2 knockdown in individual sympathetic neurons

**Figure 4.**
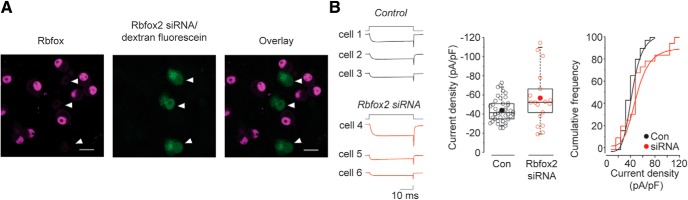
Rbfox2 is expressed in sympathetic neurons. ***A***, Representative fluorescence image of dissociated cultured neurons from SCG. Three cells (white arrows) in image shown were injected with Rbfox2 siRNA and dextran fluorescein (green) 24 h before all cells were fixed and exposed to anti-Rbfox2 (magenta). siRNA injected cells show little anti-Rbfox2 signal (white arrows), while control cells (not injected) show strong Rbfox2 expression localized to cell nuclei (scale bars, 20 μm). ***B***, left, Traces of calcium currents induce by voltage step to +20 mV from three different SCG neurons not injected (control, cells 1-3) and three neurons injected with dextran/fluorescein and Rbfox2 siRNA (cells 4-6). Middle, Average current density (filled symbol), median (horizontal line), 25th-75th interquartile range (box), and whiskers (range) are shown for calcium currents in control (not injected) neurons (*n* = 41) and in neurons injected 24 h prior with Rbfox2 siRNA (*n* = 22). Mean (median) ± SE in pA/pF were for control: 43.99 (41.6) ± 1.9; and for SCG siRNA injected: 56.61 (52.13) ± 6.75. Right, Cumulative frequency plot for charge density measured from control and Rbfox2 siRNA injected SCG neurons.

We targeted Rbfox2 mRNAs with siRNAs in cultured SCG neurons as a way to manipulate levels of Rbfox2 expression and, consequently, inclusion of e18a in Cav2.2 mRNAs. To observe the effectiveness of siRNA-mediated knockdown, we used a pan Rbfox antibody that recognizes all Rbfox proteins (RRM; Gehman et al., 2011) and observed strong immunofluorescence in nuclei of SCG neurons (as expected for a splicing factor; Fig. 4*A*). The anti-Rbfox signal was completely eliminated in individual neurons injected with Rbfox2 siRNA (coinjected with dextran fluorescein dye; Fig. 4*A*). Together with data shown in Figures 2, 3, our results demonstrate that Rbfox2 expressed in F11 cells and in sympathetic neurons regulates e18a splicing.

Rbfox2 knockdown should shift the balance of Ca_V_2.2 channels toward e18a-containing and away from e18a-lacking therefore, as a first step to test the impact of e18a on endogenous Ca_V_2.2 currents, we analyzed the effect of Rbfox2 knockdown on calcium channel activity. We used whole-cell recording to compare Ca_V_ currents evoked by voltage steps from -80 mV to +20 mV in 41 control neurons (not injected) and 22 neurons injected 3-4 d earlier with Rbfox2 siRNA (Fig. 4*B*). Ca_V_2.2 underlies ∼60-75% of the whole-cell Ca_V_ current in sympathetic neurons of mouse SCG (see below; Hirning et al., 1988). Whole-cell Ca_V_ current densities were variable across neurons, but Ca_V_ currents were generally larger in SCG neurons injected with Rbfox2 siRNA as compared to noninjected neurons (Fig. 4*B*, Mann-Whitney rank sum test, *p* = 0.06^d^ at 0 mV). The large variability of Ca_V_ current densities and the presence of neurites in 3-4 d neuronal cultures, which introduced space clamp errors, complicated our ability to draw definitive conclusions. Moreover, Rbfox2 controls splicing of pre-mRNAs other than Ca_V_2.2 in SCG neurons that may also influence Ca_V_ currents. Therefore, to test the impact of e18a on Ca_V_2.2 currents directly we used two complementary, independent approaches: (1) cell lines transiently expressing either e18a-Ca_V_2.2 or Δ18a-Ca_V_2.2 clones; and (2) sympathetic neurons from genetically modified mice that express either only e18a-Ca_V_2.2 or only Δ18a-Ca_V_2.2 channels.

### E18a is associated with larger Ca_V_2.2 currents in cell lines transiently expressing Ca_V_2.2 clones

We expressed e18a-Ca_V_2.2 or Δ18a-Ca_V_2.2 channel clones in tsA201 cell lines together with subunits Ca_V_α_2_δ_1_ and Ca_V_β_3_ or Ca_V_β_2_, which are necessary for membrane trafficking and to reconstitute features of channel gating. These Ca_V_β subunits were chosen to most closely match endogenous Ca_V_2.2 complexes in sympathetic neurons. Ca_V_β_3_ associates with >50% of Ca_V_2.2 protein in brain (Castellano et al., 1993) and Ca_V_β_3_ and Ca_V_β_2_ are both essential for the expression of functional Ca_V_2.2 channels in sympathetic neurons (Namkung et al., 1998).

**Figure 5.**
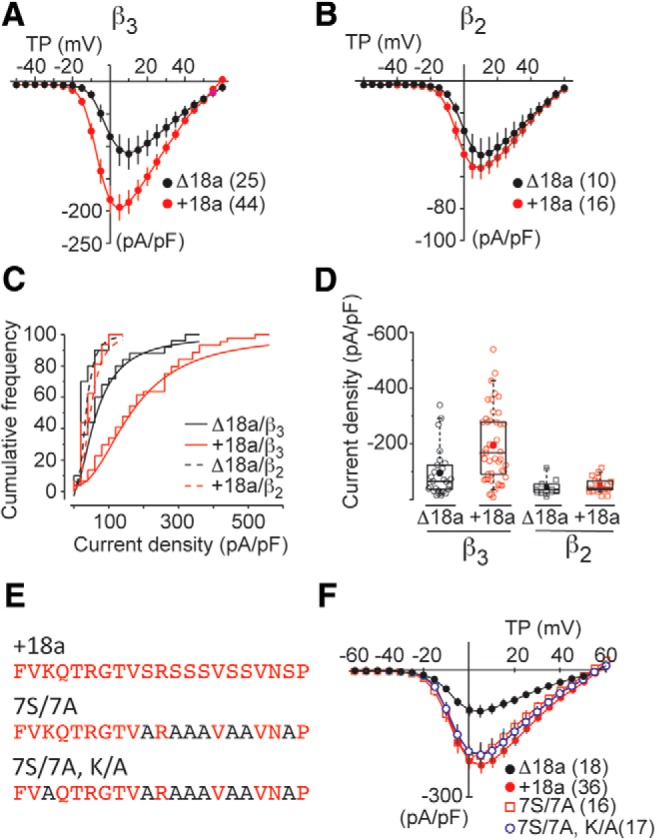
Larger current density in cells expressing e18a-Ca_V_2.2 compared to Δ18a-Ca_V_2.2. ***A***, Peak Ca_V_2.2 current−voltage relationships in tsA201 cells expressing Δ18a-Ca_V_2.2 or e18a-Ca_V_2.2 with Ca_V_β_3_ and Ca_V_α_2_δ-_1_ subunits. Values are mean ± SE and *N* values are shown in parentheses. Data were compiled from multiple different transfections and gathered by three different experimenters. Individual current−voltage relationships were fit by Boltzmann-linear functions and Boltzmann activation midpoint (V_1/2_) and slope (*k*) estimated. V_1/2_ ± SE (mV); Δ18a-Ca_V_2.2: -1.3 ± 0.9; e18a-Ca_V_2.2: -4.8 ± 0.6 (Student’s unpaired *t* test, *p* = 0.0001^e^). *k* ± SE (mV); Δ18a-Ca_V_2.2: 5.2 ± 0.5; e18a-Ca_V_2.2 = 4.4 ± 0.25. ***B***, Ca_V_2.2 current−voltage relationships in cells expressing Δ18a-Ca_V_2.2 or e18a-Ca_V_2.2 with Ca_V_β_2_ and Ca_V_α_2_δ-_1_. Values are average ± SE. ***C***, Cumulative frequency plots of peak Ca_V_2.2 current densities measured at 5 mV in cells expressing Δ18a-Ca_V_2.2 or e18a-Ca_V_2.2 with either Ca_V_β_2_ or Ca_V_β_3_. Bin size was 25 pA/pF for all plots. ***D***, Peak Ca_V_2.2 current density values are shown for individual cells (open symbols), mean (filled symbol), median (horizontal line), 25th-75th interquartile range (box), and whiskers (range) in tsA201 cells expressing Δ18a-Ca_V_2.2 or e18a-Ca_V_2.2 with Ca_V_β_3_ and Ca_V_β_2_. Mean (median) ± SE in pA/pF at 5 mV were for Δ18a-Ca_V_2.2/Ca_V_β_3_: 95.9 (66.1) ± 17.1; e18a-Ca_V_2.2/Ca_V_β_3_: 194.5 (167.7) ± 19.4; Δ18a-Ca_V_2.2/Ca_V_β_2_: 42.9 (32.3) ± 9.4; e18a-Ca_V_2.2/Ca_V_β_2_ 53.9 (41.6) ± 7.1 (one-way ANOVA on ranks, Bonferroni corrected *p* = 0.0001^f^). ***E***, Amino acid sequence of wild-type e18a exon (+18a), and two mutants. Seven serines were substituted with alanines (7S/7A) in both mutants; one mutant contains an additional lysine to alanine substitution (7S/7A, K/A). ***F***, Peak Ca_V_2.2 I-V relationships in cells expressing Δ18a-Ca_V_2.2, e18a-Ca_V_2.2 WT, e18a-Ca_V_2.2 7S/7A, and e18a-Ca_V_2.2 7S/7A, K/A. Mean and SE for each condition and *N* values in parentheses. Mean (median) ± SE in pA/pF at 5 mV for WTe18a-Ca_V_2.2: -248.64 (232.1) ± 24.6; 7S/7A: -203.8 (250.3) ± 25.9; 7S/7A,K/A: -190.2 (170.2) ± 29.9 (one-way ANOVA on ranks, Bonferroni corrected, p_WT:7S/7A_ = 0.262^g^ and one-way ANOVA on ranks, Bonferroni corrected, p_WT: 7S/7A,K/A_ = 0.213^g^).

We compared peak Ca_V_2.2 current−voltage relationships of e18a-Ca_V_2.2 (44 cells) and Δ18a-Ca_V_2.2 (25 cells). Ca_V_2.2 currents were evoked from a holding potential of -100 mV to remove steady-state inactivation (Pan and Lipscombe, 2000; Thaler et al., 2004). Ca_V_2.2 current densities in cells expressing e18a-Ca_V_2.2/Ca_V_β_3_ were consistently larger compared to those from cells expressing Δ18a-Ca_V_2.2/Ca_V_β_3_ (Fig. 5*A*). At the peak of the current−voltage relationship, e18a-Ca_V_2.2/Ca_V_β_3_ current densities were approximately twice as large on average, compared to Δ18a-Ca_V_2.2/Ca_V_β_3_ currents (Fig. 5*D*, one-way ANOVA on ranks, Bonferroni corrected *p* = 0.0001^f^). This difference in overall Ca_V_2.2 current density is also apparent when cumulative distributions of current densities for each clone are compared (Fig. 5*C*, currents were evoked by voltage steps to 5 mV). Individual I-V plots were fit to a Boltzmann-linear function and V_1/2_ and *k* values were estimated for each condition. Ca_V_2.2 currents activated at slightly more negative voltages in cells expressing e18a-Ca_V_2.2 compared to those in cells expressing Δ18a-Ca_V_2.2 (Fig. 5*A*, Student’s unpaired *t* test, *p* = 0.0001^e^) but the slope of activation curves were in distinguishable (Fig. 5*A*, Student’s unpaired *t* test, *p* = 0.89^e^). Although not highlighted, previous studies in cell lines also show a consistent trend toward larger Ca_V_2.2 currents in cells expressing e18a-Ca_V_2.2 compared to Δ18a-Ca_V_2.2 clones (Thaler et al., 2004) as well as slight differences in voltage-dependence of activation (Pan and Lipscombe, 2000).

The e18a sequence contains seven serines, a subset of which, we hypothesized, may be targets of serine/threonine kinases with the potential to influence membrane trafficking. However, Ca_V_2.2 currents in cells expressing e18a-Ca_V_2.2/Ca_V_β_3_ in which all seven serines were replaced by alanines (7S/7A) were not consistently different from wild-type e18a-Ca_V_2.2/Ca_V_β_3_ currents (Fig. 5*E*,*F*, one-way ANOVA on ranks, Bonferroni corrected, p_WT:7S/7A_ = 0.262^g^). We performed an additional mutation, replacing the one lysine in the e18a sequence with alanine, but this construct (7S/7A, K/A) generated Ca_V_2.2 currents of similar overall size to e18a-Ca_V_2.2 (Fig. 5*E*,*F*, one-way ANOVA on ranks, Bonferroni corrected, p_WT: 7S/7A,K/A_ = 0.213^g^). Therefore, although we have not yet determined the mechanism underlying the larger current amplitude in cells expressing e18a-Ca_V_2.2/Ca_V_β_3_, we can eliminate a critical role for lysine or serine residues encoded by e18a.

We also compared e18a-Ca_V_2.2 (16 cells) and Δ18a-Ca_V_2.2 (10 cells) in cells expressing Ca_V_β_2_. Overall, Ca_V_2.2/Ca_V_β_2_ current densities were ∼50% of those in cells coexpressing Ca_V_β_3_ independent of e18a inclusion (Fig. 5, compare *A-D*). E18a-Ca_V_2.2/Ca_V_β_2_ currents were on average only slightly larger compared to Δ18a-Ca_V_2.2/Ca_V_β_2_ and with reduced statistical certainty as compared to experiments with Ca_V_β_3_ (Fig. 5*B*,*D*, one-way ANOVA on ranks, Bonferroni corrected, *p* = 0.11^f^).

### E18a-Ca_V_2.2 is associated with larger Ca_V_2.2 currents in sympathetic neurons

**Figure 6.**
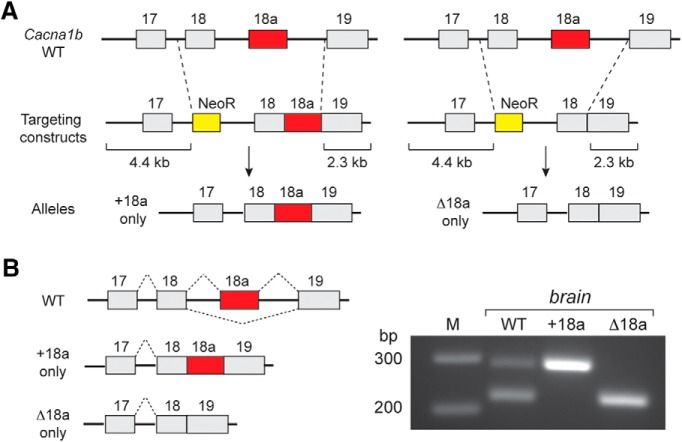
Mutation strategy to restrict exon choice at e18a locus of mouse *Cacna1b* gene. ***A***, Schematic illustrates homologous recombination strategy used to generate two mouse strains that express either +18a-only (*Cacna1b^tm3.1Dili^*) or Δ18a-only (*Cacna1b^tm4.1Dili^*) Ca_V_2.2 transcripts. Targeting constructs contained 4.4 kb (left) and 2.3 Kb (right) recombination arms, and a NeoR cassette was used to select for successful recombination events in embryonic stem cells. ***B***, left, Splicing patterns in *Cacna1b* pre-mRNA of WT, +18a-only, and Δ18a-only mice. Right, RT-PCR products from WT, +18a-only Ca_V_2.2, and Δ18a-only Ca_V_2.2 mouse brain. Primers were located in e18 and e19 as described in Figure 1. Expected bands were observed: two different size PCR products corresponding to +18a and Δ18a from WT, one band corresponding to +18a from +18a-only samples, and one band corresponding to Δ18a from Δ18a-only samples.

Several cellular factors can influence Ca_V_2.2 channel function emphasizing the importance of assessing the function of e18a-Ca_V_2.2 and Δ18a-Ca_V_2.2 channels in their native environment and in association with Ca_V_β subunits of sympathetic neurons. We generated two novel strains of mice that either expressed only e18a-Ca_V_2.2 (+e18a, *Cacna1b^tm3.1Dili^*) or only Δ18a-Ca_V_2.2 channels (Δe18a, *Cacna1b^tm4.1Dili^*) by eliminating the splice site and introns and replacing the endogenous locus with one of two large exons containing e18-e19 (Δ18a) or e18-e18a-e19 (+18a) in frame, with no intervening introns (Fig. 6*A*). RT-PCR analyses of brain shows that this strategy was successful; wild-type mice express both e18a-Ca_V_2.2 and Δ18a-Ca_V_2.2 mRNA isoforms whereas only e18a-Ca_V_2.2 mRNAs were amplified from +18a mice and only Δ18a-Ca_V_2.2 mRNAs were amplified from Δ18a mice (Fig. 6*B*). Mice homozygous for e18a-Ca_V_2.2 and Δ18a-Ca_V_2.2 were viable and fecund (see Materials and Methods; Table 1).

**Table 1.** Mice homozygous for e18a-Ca_V_2.2 and 18a-Ca_V_2.2 were viable and fecund

Cross (het X het)	**Cacna1b*^*tm3(*^*^e18a^*^*)DiLi*^*	**Cacna1b*^*tm4(*^*^Δe18a)^*^*DiLi*^*
Litters	32	42
Progeny	259	322
Progeny/litter	8.1	7.7
Homozygous	60	82
Heterozygous	137	152
Wild type	61	88
Homozygous dead at weaning	1	1
Ratio of progeny observed (mut/mut:wt/mut:wt/wt)	0.9:2.2:1	0.9:1.7:1
Ratio of progeny expected (mut/mut:wt/mut:wt/wt)	1:2:1	1:2:1

**Table 2.** Table of statistics

	Data structure	Type of test	CI_95%_ (bootstrapping with replacement)
a	Normal	Student’s unpaired *t* test	Nontransfected:18.0 – 27.0%100 nM siRNA to Rbfox2: 53.5 – 63.3%
b	Normal	One-way ANOVA/Bonferroni corrected	Nontransfected:18.0-27.0%CycB:15.8-24.8%Nontargeting siRNA:22.7-29.3%
c	Normal	Student’s *t* test unpaired	% Rbfox2 relative to inputAdult:-0.05 to 0.42 (*n* = 3)P8-P11:0.23 to 0.86 (*n* = 3)
d	Not normal	Mann-Whitney rank sum	Control:40.28-47.7 pA/pFSCG siRNA injected:43.4-69.84 pA/pF
e	Normal	Student’s *t* test unpaired	V_1/2_ Δ18a/Ca_V_β_3_:-3.1 to -0.5 mV18a/Ca_V_β_3_:-5.6 to -3.6 mV*k* Δ18a/Ca_V_β_3_:4.2 to 6.2 mV18a/Ca_V_β_3_:3.9 to 4.9 mV
f	Not normal	One way ANOVA on ranks, Bonferroni corrected	Δ18a/Ca_V_β_3_:62.4-129.4 pA/pFε18a/Ca_V_β_3_:156.5-323.2 pA/pFΔ18a/Ca_V_β_2_:25.0-60.8 pA/pFε18a/Ca_V_β_2_:41.0-66.8 pA/pF
g	Not normal	One-way ANOVA on ranks, Bonferroni corrected	WTe18a:200.4-296.8 pA/pF7S/7A:153.0-254.6 pA/pF7S/7A, K/A:133.0-248.7 pA/pFΔ18a: 54.7-145.4 pA/pF
h	Not normal	Mann-Whitney rank sum	Δ18a-only:55.2-67 pA/pFe18a-only:59.7-86.9 pA/pF
i	Normal	Student’s *t* test unpaired	N-type current:Δ18a-only:-30.0 to -48.8 pA/pFe18a-only:-52.8 to -73.4 pA/pFNon-N-type current:Δ18a-only:-26.6 to -35.4 pA/pFe18a-only:-23.3 to -31.7 pA/pF
j	Normal	Student’s *t* test unpaired	V_1/2_ Δ18a-only: -31.05 to -14.05 (mV)e18a-only: -29.4 to -11.6 (mV)*k* Δ18a-only: -21.6 to -18.3 (mV)e18a-only: -19.97 to -13.57 (mV)

**Figure 7.**
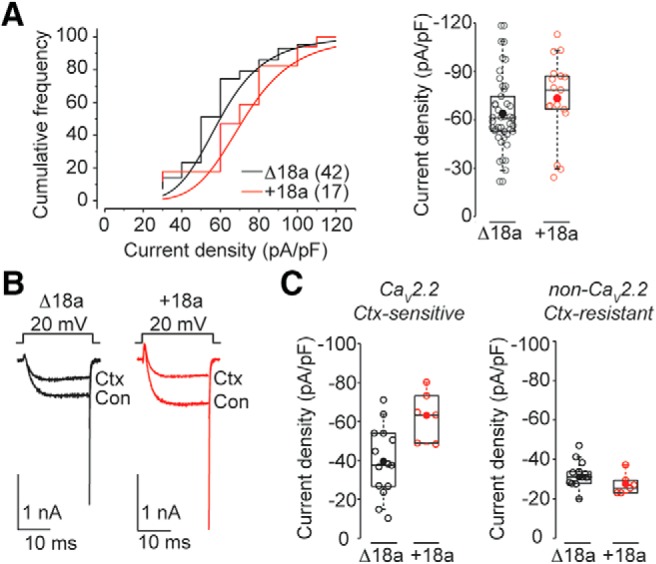
Ca_V_2.2 currents in +18a neurons are larger compared to Δ18a-only neurons. ***A***, left, Cumulative frequency plots of peak Ca_V_ current density recorded at 20 mV in SCG neurons from Δ18a-only and +18a-only mice. *Right*, peak Ca_V_ current density at 20 mV (pA/pF). Mean (median) values ± SE were for Δ18a-only: 61.54 (60.1) ± 3.24; +18a-only: 73.3 (78.5) ± 6.94 (Mann-Whitney rank sum test *p* = 0.035i^h^). ***B***, left, Ca_V_ currents evoked by voltage steps to +20 mV from -80 mV in the absence (Con) and presence of 2 μM ω-conotoxin GVIA (Ctx) in acutely dissociated SCG neurons. ***C***, Ca_V_2.2 (Ctx-sensitive; left) and non-Ca_V_2.2 (Ctx-resistant; right) currents in SCG neurons from Δ18a-only (black) and +18a-only (red) mice. Values for Ca_V_2.2 current density mean (median) ± SE for Δ18a-only: -39.43 (-37.8) ± 4.8 pA/pF; +18a-only: 63.1 (-64.0) ± 5.2 (Student’s *t* test, unpaired *p* = 0.009^i^). Right, Values for non-Ca_V_2.2 current density mean (median) ± SE for Δ18a-only: -31.1 (-31.0) ± 2.3 pA/pF; e18a-only: -27.5 (-26.2) ± 2.2 (Student’s *t* test, unpaired *p* = 0.353^i^). Experimenter was blind to genotype until after all recordings and analyses were complete. All box plots: values shown are for individual cells (open symbols), mean (filled symbol), median (horizontal line), 25th-75th interquartile range (box), and whiskers (range).

We compared Ca_V_ currents recorded from acutely dissociated adult sympathetic neurons from +18a and Δ18a mice and found that the total Ca_V_ current in sympathetic neurons evoked by test pulses to +20 mV was on average ∼20% larger in neurons from +18a compared to Δ18a mice (Fig. 7*A*, Mann-Whitney rank sum test *p* = 0.035^h^). The whole-cell Ca_V_ current in sympathetic neurons is comprised of Ca_V_2.2 and non-Ca_V_2.2 subtypes, but ∼60-65% originates from the gating of Ca_V_2.2 channels. To isolate Ca_V_2.2 from non-Ca_V_2.2 current, we used ω-conotoxin GVIA subtraction to identify and isolate a pure Ca_V_2.2 current (see Materials and Methods; Andrade et al., 2010; Fig. 7*B*). We found that the amount of Ca_V_ current arising from Ca_V_2.2 channels was ∼60% larger, on average, in sympathetic neurons from +18a mice as compared to Δ18a mice (Fig. 7*B*, Student’s *t* test, unpaired *p* = 0.009^i^). The non-N-type component of the Ca_V_ current was not consistently different in sympathetic neurons isolated from the two genotypes (Fig. 7*C*, Student’s *t* test, unpaired *p* = 0.353^i^). Thus, the larger overall Ca_V_ current in +18a mice originates from differences in Ca_V_2.2 current levels.

### Inactivation properties of +18a and Δ18a Ca_V_2.2 currents in sympathetic neurons are not different

**Figure 8.**
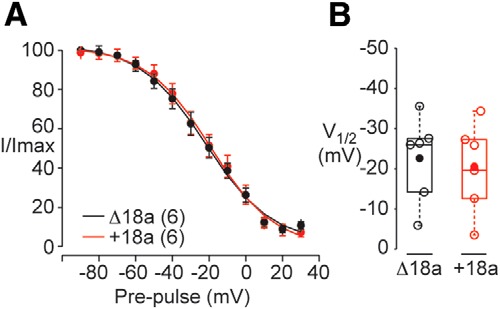
Inactivation of Ca_V_2.2 currents in sympathetic neurons is indistinguishable in Δ18a-only and +18a-only mice. ***A***, Averaged, normalized, steady-state inactivation curves generated from sympathetic neurons from Δ18a-only and +18a-only mice. Recordings were performed in the presence of ω-agatoxin IVA (50 nM), and isradipine (10 μM) to block Ca_V_2.1 and Ca_V_1 currents, respectively. Ca_V_2.2 currents were inactivated by 3-s prepulses to different membrane voltages (between -90 and + 20 mV in 10-mV increments) and then currents evoked by 25-ms test pulses to 20 mV to measure Ca_V_2.2 channel availability. Curves were fit by Boltzmann functions and V_1/2_ values estimated. ***B***, V_1/2_ values from individual cells (open symbols), mean (filled symbol), median (horizontal line), 25th-75th interquartile range (box), and whiskers (range). Mean (median) values ± SE in mV were for Δ18a-only: -22.5 (-26.1) ± 4.3, and e18a-only mice**:** -20.1 (-22.7) ± 4.5.

Ca_V_2.2 channels in sympathetic neurons are thought to primarily associate with Ca_V_β_3_ or Ca_V_β_2_ subunits (Lin et al., 1996; Namkung et al., 1998; Heneghan et al., 2009). Based on previous analyses of Δ18a-Ca_V_2.2 and e18a-Ca_V_2.2 clones expressed with Ca_V_β_3_ or Ca_V_β_2_ subunits (Pan and Lipscombe, 2000), we expect inactivation properties of Ca_V_2.2 channels in sympathetic neurons from Δ18a-only and e18a-only mice to be similar. Nonetheless, to be certain, we compared inactivation properties of e18a-Ca_V_2.2 and Δ18a-Ca_V_2.2 currents in their native environment in sympathetic neurons. We inhibited Ca_V_1 and Ca_V_2.1 currents with a combination of isradipine (10 μM) and ω-agatoxin IVA (50 nM) and used a two-step protocol to measure steady-state inactivation of Ca_V_2.2 currents: 3 s prepulses to different membrane voltages between -90 and 20 mV, followed by a test pulse to + 20 mV (Fig. 8*A*). The inactivation properties of e18a-Ca_V_2.2 and Δ18a-Ca_V_2.2 currents in sympathetic neurons were indistinguishable (Fig. 8*B*, V_1/2_ Student’s *t* test, unpaired *p* = 0.75^j^).

Our data also add independent evidence that the properties of Ca_V_2.2 currents in sympathetic neurons are most similar to those of Ca_V_2.2/Ca_V_β_2_ and Ca_V_2.2/Ca_V_β_3_ channels. The mid-point of the steady state inactivation curve of Ca_V_2.2 currents recorded in sympathetic neurons is approximately -20 mV (Fig. 8*B*; 1 mM Ca^2+^ as charge carrier), a value that is more consistent with voltage-dependent inactivation profile of Ca_V_2.2 currents associated with Ca_V_β_2_ or Ca_V_β_3_ subunits, see (Pan and Lipscombe, 2000; Thaler et al., 2004).

## Discussion

Alternative splicing is extensive in mammalian nervous systems (Fagnani et al., 2007). Multiexon ion channel pre-mRNAs are targets of neuronal splicing factors, which are proposed to individualize exon composition of ion channel mRNAs according to cell needs. While the global importance of alternative pre-mRNA splicing has been amply demonstrated, our study is one of only a few to demonstrate a functional consequence of a single alternatively spliced exon, and to identify the RNA binding protein that controls its expression.

### Rbfox2 suppresses e18a inclusion

Our study focuses on e18a, an alternatively spliced cassette exon that is a conserved feature of human, rat and mouse *Cacna1b* genes. Alternative exons that are similarly spliced among multiple species may have functional importance that has been preserved evolutionarily (Modrek and Lee, 2003). We show that the pattern and approximate frequency of e18a inclusion in mRNAs of human, rat and mouse tissues are remarkably similar, suggestive of a common regulatory mechanism. It is likely that Rbfox2 represses e18a inclusion during alternative splicing of Ca_V_2.2 pre-mRNAs in these species, consistent with the conserved Rbfox2 binding motif upstream of e18a in several vertebrates (Fig. 1; Kent et al., 2002). We also highlight the utility of using vertebrate conservation tracks, accessible through various genome browsers, as a way to identify potential conserved alternatively spliced exons that have restricted expression profiles and, consequently, that are frequently not well represented in mRNA-derived databases or annotated in genomes (Lipscombe et al., 2015).

Rbfox2 is one of a family of three RNA binding proteins (Rbfox1, 2, and 3) that coordinate splicing of a network of alternative exons important for normal development (Zhang et al., 2008; Yeo et al., 2009; Lovci et al., 2013; Jangi et al., 2014; Weyn-Vanhentenryck et al., 2014). There is substantial overlap in the binding patterns of different Rbfox proteins and although we demonstrate directly that Rbfox2 controls e18a inclusion in sympathetic neurons (CLIP-RT-qPCR), our study does not exclude roles for Rbfox1 and Rbfox3, in addition to Rbfox2, in regulating e18a expression in other cell types (Weyn-Vanhentenryck et al., 2014). Cell-specific splicing decisions can also involve other factors including second messengers, alternative splicing, and microRNAs (Chen and Manley, 2009; Venables et al., 2013; Klinck et al., 2014; Li et al., 2015; Wang et al., 2015).

### Function of e18a in Ca_V_2.2

By comparing e18a isoforms of Ca_V_2.2 in a reduced expression system, we showed that the presence of the 21 amino acids encoded by e18a in the II-III loop of Ca_V_2.2 is associated with larger Ca_V_2.2 currents, the magnitude of which depends on which Ca_V_β subunit is expressed. We also showed that the larger current phenotype associated with e18a was a feature of Ca_V_2.2 currents in sympathetic neurons of mice that we engineered to express Ca_V_2.2 channels that all contained the e18a sequence. Interestingly, although not highlighted in previous analyses of e18a-Ca_V_2.2 splice isoforms, a trend toward larger Ca_V_2.2 current amplitudes is apparent when comparing e18a-Ca_V_2.2 and Δ18a-Ca_V_2.2 currents expressed in tsA201 cells (Thaler et al., 2004). The II-III linker of Ca_V_ channels is known to influence protein trafficking to, and retrieval from the plasma membrane (Altier et al., 2002; Bernstein and Jones, 2007) findings that are consistent with our observations. Conditional deletion studies of Rbfox2 in mouse brain have shown that this splicing factor is important during development of cerebellum and Purkinje neurons; Rbfox2 CNS-null mice have disrupted dendritic arborization and abnormal migration (Gehman et al., 2012). Rbfox2 has also been implicated in cardiac development in zebrafish (Gallagher et al., 2011) and possibly in humans (Verma et al., 2016).

### Functional consequences of e18a on current amplitude depends on Ca_V_β

Ca_V_2.2 α_1_ subunits must associate with a Ca_V_β subunit to be displaced from the endoplasmic reticulum and to traffic to the plasma membrane (Bichet et al., 2000). The major site of Ca_V_β binding for ER displacement is in the I-II linker. Although secondary interaction sites of Ca_V_β with Ca_V_α_1_ subunits have been reported, these do not include the II-III linker of Ca_V_ (Walker et al., 1998; Walker et al., 1999). It is not necessary to propose that e18a interacts directly with Ca_V_β subunits. For example, Ca_V_β_2_ may limit trafficking of Ca_V_2.2 to the plasma membrane and thereby obscure any potential influence of e18a. As we report here, Ca_V_2.2/Ca_V_β_2_ currents are smaller compared to Ca_V_2.2/Ca_V_β_3_ currents recorded under the same conditions, and independent of e18a.

Numerous studies have reported interactions between the II-III linker of Ca_V_2.2 and various proteins, including synaptic vesicle proteins, syntaxin and SNAP25, as well as several second messenger pathways. In some cases these interactions are close to the site of the e18a peptide (Magga et al., 2000; Cao et al., 2004; Szabo et al., 2006). Moreover, proteins and second messengers that interact with the II-III linker have significant impact on overall Ca_V_2.2 current density (Kisilevsky et al., 2008). Therefore, it is possible that the presence of e18a either promotes or disrupts interactions with proteins that affect Ca_V_2.2 trafficking. We do not know the mechanism by which the e18a-encoding sequence promotes Ca_V_2.2 channel expression but, by alanine substitution, we have eliminated a direct contribution of seven serines and one lysine of the 21 amino acids in the 18a sequence.

Cellular mechanisms have evolved to regulate inclusion of certain exons depending on cell type and stage of development (Li et al., 2007; Licatalosi et al., 2008; Vuong et al., 2016). We present evidence that Rbfox2 repression of exon inclusion, through its binding to *Cacna1b* during splicing of Ca_V_2.2 pre-mRNA, can impact the overall contribution of Ca_V_2.2 channels to voltage-dependent calcium current in sympathetic neurons. We suggest that Rbfox2-mediated repression of e18a early in development limits the size of the Ca_V_2.2 calcium signal in sympathetic neurons of SCG.

## Synthesis

Reviewing Editor: Muriel Thoby-Brisson, CNRS UMR 5287 Université Bordeaux 2 Decisions are customarily a result of the Reviewing Editor and the peer reviewers coming together and discussing their recommendations until a consensus is reached. When revisions are invited, a fact-based synthesis statement explaining their decision and outlining what is needed to prepare a revision will be listed below. The following reviewer(s) agreed to reveal their identity: Kevin Currie

Both reviewers agree on the fact that revisions were properly done and significantly improved the paper that now represents a very important advance in the field.
